# Perspectives and Solutions from Clinical Trainees and Mentors Regarding Ethical Challenges During Global Health Experiences

**DOI:** 10.5334/aogh.2721

**Published:** 2020-03-26

**Authors:** Jennifer Kasper, Anita Mulye, Ashti Doobay-Persaud, Brittany Seymour, Brett D. Nelson

**Affiliations:** 1 Harvard Medical School, US; 2 Northwestern Feinberg School of Medicine, US; 3 Harvard School of Dental Medicine, US

## Abstract

**Background:**

Clinical trainees face challenges throughout short-term experiences in global health (STEGH) that are not routinely addressed.

**Objectives:**

Describe common professional and ethical dilemmas faced by clinical trainees and identify gaps and solutions for pre, during, and post-STEGH training and mentoring.

**Methods:**

We conducted a mixed-methods study among trainees and mentors involved in global health. The study utilized focus groups with trainees (November–December 2015) and online surveys of trainees, in-country and stateside faculty mentors (October 2016–April 2017).

**Results:**

85% (17/20) of students reported feeling prepared for their STEGH; however, 59% (23/39) of faculty felt students were unprepared. A majority of both students (90%) and faculty (77%) stated students would likely experience an ethical dilemma during STEGH. Major themes relating to meaningful global health work were elucidated: personal and inter-professional skills; interpersonal networks and collaboration; and awareness of power dynamics and bias.

**Conclusions:**

The most common challenges faced by trainees during STEGH related to leadership, bias, ethics and interprofessional collaboration. Redirecting trainee energies from a focus on ‘doing’ and deliverables to attitudes (e.g., humility, professionalism) that cultivate personal and professional growth will help create lifelong global health learners and leaders.

## Introduction

The number of global health opportunities presented to clinical trainees in high-income countries (HICs) continues to grow at a rapid rate [1]. These experiences provide a number of meaningful benefits to trainees, including clinical knowledge, enhanced compassion, empathy, and cultural sensitivity in patient care [8,9]. Multiple studies also demonstrate that global health electives increase a trainee’s understanding of macroeconomic issues, public health systems, health inequity, and the importance of care for underserved populations [15].

However, a growing body of literature also elucidates challenges faced by trainees from HICs working abroad during short-term experiences in global health (STEGH) [16]. The most common themes include ethical dilemmas, potential burden on the host community, cultural differences, and clinical challenges. A recent study identified four major ethical themes faced by students: cultural differences (e.g., informed consent, autonomy), professional issues (e.g., power dynamics, perceived corruption, training of local staff), limited resources (e.g., scope of practice, logistical scarcities), and personal moral development (e.g., moral distress, humility, self-awareness) [17]. Another study found that including an ethics workshop prior to a global health elective for medical students improved students’ self-rating of preparedness to manage ethical dilemmas [3]. Even though a number of residency programs and organizations (e.g., Consortium of Universities for Global Health and the American Academy of Pediatrics) have created structured global health guidelines and competencies to address the challenges most commonly faced by medical students and faculty from HICs while working abroad, these need to be put into practice [3,6,18,20]. In addition, trainees would benefit from ongoing mentorship before, during, and after their STEGH to provide more opportunities for critical reflection and transformative learning [5].

While a number of published case reports and clinical vignettes highlight near misses and actualized ethical dilemmas, there is a relative dearth of robust examinations of and data collection about the character and prevalence of ethical issues faced by clinical trainees working in diverse geographic locations, studying various health topics, and serving a range of populations [7,11]. In-country mentors’ voices and wisdom need to be elevated and incorporated in a longitudinal way [12]. Instilling global health ethics training is timely and crucial, and requires vigilance and supervision [14].

The aim of this multi-modal study is to describe, through quantitative and qualitative analysis, perspectives and potential solutions in global health training across the continuum (i.e., pre, during and post-STEGH) as identified by clinical trainees and mentors at a medical and dental school in a HIC. These may help prevent, mitigate, or resolve ethical challenges that often occur in global health experiences and enhance collaborative, effective, ongoing global health work.

## Methods

The study included trainee focus group discussions and online surveys of trainees and faculty at Harvard Medical School (HMS), Harvard School of Dental Medicine (HSDM) (Boston, MA, USA) or who mentored student global health electives. A purposive sampling strategy was utilized in which an email was sent to all trainees who had completed a school-approved summer global health experience between 2012 and 2015 and to all of their faculty mentors. Authors JK and BS developed a semi-structured focus group guide (See Appendix A), the preliminary findings of which informed the creation of online trainee and faculty surveys containing open- and closed-response questions, which utilized skip-logic questioning about pre-trip preparation, bias, culture shock, reverse culture shock, ethical challenges, and how to enhance preparation and the overall experience before, during, and after a STEGH. Audio recordings of the focus group discussions were transcribed verbatim. Two authors independently reviewed the transcripts and survey open-responses and generated initial codes by hand. These codes were refined into a consolidated list of codes that were sorted into potential themes, followed by collation of all relevant coded data extracts within identified themes using inductive logic without employing a specific theoretical perspective. The authors then mined the data for illustrative quotes for each theme. Throughout coding and theme generation, a third author provided regular input and served as the arbiter if discrepancies arose from coding. The study received exemption from the Harvard Medical School institutional review board.

## Results

A total of 15 students (13 medical and 2 dental) participated in one of three 90-minute focus groups during November and December 2015; 13 (87%) of participants were female, and all were third-year students. The authors believe they achieved thematic saturation during data collection as, by the end of the discussions, they were no longer receiving novel responses.

Distinct online surveys for trainees and faculty were created based on preliminary analysis of the themes identified from the focus groups. A total of 116 students who had completed a global health project during the summers of 2012–15 received an email inviting them to complete an online survey. Twenty (17%) students completed the survey: 12 (60.0%) were female, and there were 16 (80.0%) third-year, three (15.0%) fourth-year, and one (5.0%) fifth-year student. Students stated that the top three most useful aspects of pre-trip preparation were conversations with on-site mentors (16, 80%), HMS/HSDM mentors (15, 75%), and other students (10, 50%). While 17 (85%) reported they were prepared or extremely prepared for their global health experience, 13 (65%), 12 (60%), and nine (45%), reported they wish they had more expertise in a) site-specific practices, culture, and context; b) research skills (e.g., quantitative and qualitative methods, study design, and analytic skills); and c) leadership, diplomacy, and project management, respectively, prior to their STEGH.

Less than 10% stated that they wish they had more expertise in ethics, human rights, social determinants of health, interpersonal communication and teamwork, culture and reverse culture shock, even though 18 (90%) reported it was likely or extremely likely that a student will experience an ethical dilemma while participating in a global health experience; nine of 16 students (56%) reported they experienced an ethical dilemma during their global health experience; and half of these reported they were unprepared to appropriately manage it. In addition, 11 (55%), eight (40%), and five (25%) students experienced bias in themselves, culture shock, and reverse culture shock, respectively, during their STEGH. When asked to elaborate on their ethical dilemmas, their comments related to providing financial support to in-country people, study design and participant consent, practicing beyond the scope of one’s training, and police corruption. When asked to rank their ability to identify ways a student can prepare for a dilemma they might face, or list strategies for managing conflicting impulses, emotions, and motivations, half said “fair or poor.” When asked about a post-STEGH debrief, only one-third of students stated they had one, and they stated they would like to see the following: informal, discussions with someone who has done global health work to discuss next steps personally and professionally; how to stay involved in a project while stateside and during medical training; and advice navigating ethical dilemmas – specifically, what they would do differently. Students recommended ways their institution could further support students in developing their capabilities for productive engagement in global health: regular check-ins with mentors, conversations with students doing similar work in similar settings, more training in project design, support with IRB process, and discussions of ethical issues.

Ninety-six HMS and HSDM U.S.-based and in-country faculty members who mentor students in STEGHs were sent an email inviting them to complete an online survey. Forty faculty (42%) responded: 21 (52.5%) were HMS/HSDM mentors, nine (22.5%) were in-country mentors, 10 (25.0%) were both. Sixteen (40.0%) had been mentoring trainees for more than 10 years, eight (20.0%) for 5–10 years, and 16 (40.0%) for 1–5 years. Twenty-one (52.5%) had more than 10 years of global health experience, 13 (32.5%) had 5–10 years, and two (5.0%) had less than five years. When asked if they thought students were prepared academically to conduct global health work, twenty-three (59%) said students were unprepared or very unprepared. However, the majority (37, 95%) stated that students interacted and worked effectively or very effectively with other researchers and staff at their organizations. Similar to the student responses, the majority (20, 77%) stated that students experienced ethical dilemmas during their global health experience that related to the following: local mores, country specific laws (e.g., abortion is illegal), short-term gains at the expense of long-term visibility, IRB (time to get approval, trying to short cut community approval), imbalance of personal resources between student and local staff, lack of supplies to save lives that would have been saved in US, inappropriate staff behavior. Despite their busy schedules and time constraints, the majority (92%) of mentors provided feedback to students frequently or always, and the majority (94%) said students were able to receive feedback and adjust their behavior accordingly. Faculty mentors were asked to name one benefit and one unintended consequence of student global health experiences to their institution. Their responses focused on student-specific attributes, work load, cross-cultural exchange, institutional visibility, and future collaborations (Table 1).

**Table 1:** Faculty Mentor Observations about Benefits and Unintended Consequences of Student STEGH for In-Country Institutions.

Benefits	Unintended Consequences

Help get the work done, increase capacity Become future staff members	Time away from mentoring local (in-country) staff
Provide inspirational enthusiasm. It is fun to re-experience things we know well through the eyes of students experiencing them for the first time.	When a trainee is not a good fit, they can be quite draining in terms of mentorship time and emotional energy to the team. One person’s struggle (which pales in comparison to the struggles of many local staff members) can overpower the greater team needs, and in worst case scenarios can break trust and have implications for broader partnership work and potential for future students.
Are more sensitive and aware clinicians	
Enable greater cross-cultural exchange	
Open door for more collaboration and networking	
Raise institution’s profile/social capital	

Interestingly, overlapping themes emerged when faculty mentors were asked both to name a goal in mentoring students and recommend topics to cover during global health training: discuss and debate (more “book knowledge” is insufficient professionalism), context and local culture, potential benefits and perils of STEGH, setting expectations, and structuring a STEGH that is collaborative and addresses local priorities (Table 2).

**Table 2:** Faculty Mentor Recommendations Regarding Themes for Discussion During Mentoring and Training.

Theme	Comments

Context and local culture	Expand their world view
	Have them respect the culture and local beliefs, even if they disagree
	[Our goal is] that they leave our program with a MUCH better contextual approach to global healthcare and clinical practice generally that will make them much more effective at (and motivated to do) future global service work and better clinical practitioners in their home careers
	Make students aware of the global burden of unnecessary suffering
Setting and managing expectations	Realistic expectations (about how much progress can be made in short time period)
	Defining success (learning about others cultures and building trust with a team are huge successes that are undervalued)
Understanding impact of STEGH	Not all global health work is effective and some is destructive and counterproductive
	There has been lots of efforts wasted in global health
	They need to understand why failures occur in this field
	Understand impact (and in some cases, burden) that they impose on their hosts
	Getting them to understand our mantra of 3C’s in global health – co-creation, collaboration, and capacity-building — is a great first step
Local priorities and collaboration	Need to let work be informed by local knowledge and priorities
	Understand that all project development that happens in the classroom (which can be an important intellectual exercise) needs to be revisited honestly in the field with the local team that has a much deeper understanding of the work and ultimately will drive and continue the work moving forward
	Foster understanding of importance of collaborating with local partners

The major themes for enhancing STEGHs that emerged from the analysis of trainees and mentors responses included the following: (1) global health work requires key personal attributes that include humility, cultural sensitivity, openness, and flexibility; (2) interpersonal networks and collaboration at home and abroad are necessary at all stages of a global health experience – prior to departure, while in country, and upon return; (3) one major pitfall in global health work is a lack of appreciation of power dynamics (e.g., assigning priority to a trainee’s project over local needs and priorities); (4) bias exists everywhere and in multiple forms, and awareness of bias is critical; (5) reverse culture shock and guilt about inequities can be pervasive; and (6) ethical challenges are prevalent (Table 3).

**Table 3:** Themes and Student and Faculty Mentor quotes regarding STE GHs (extracted from focus group discussions and open-response survey questions).

Theme	Students’ Illustrations	Faculty Mentors’ Illustrations

Global health work requires key personal attributes that include humility, cultural sensitivity openness, flexibility	“I don’t think there’s a globally competent person […] when you go abroad to any country whether it’s in Africa or South Asia or South America, I don’t think you can be actually competent in any culture. But I think you can bring with you a knowledge about that culture and humility and openness to 1earn about the culture.” “I would say not that a person has to have incredibly deep knowledge of a million different cultures, but that they are open and willing to listen and understand other cultures.” “…realizing that when we do go to other people’s houses, their countries, their institutions, as much as we’re coming from a place of great resources with great healthcare capacity there’s a way that we have to conduct to ourselves that allows us to achieve the right aims and that you have to do it respectfully and it takes time.” “Someone who simultaneously can acknowledge incredible difference but also the same humanity and that the people that you’re working with are real people and are equal to you in what they deserve and how they deserve to be treated but at the same time may have totally different backgrounds and approaches to problems, which I feel is a hard balance to strike.”	“[Global health professionalism involves a] deep appreciation of BOTH the values, attitudes, beliefs, and resources of other social groups and communities AND the boundaries and potential impact (both positive and negative) of one’s own values, attitudes, beliefs, and resources.” Not easy to appreciate [cultural differences] by simple “book learning.” When they [students] travel to a new country for a global health experience, very rarely do I hear them ask questions about local beliefs, values, desires (rather than give their advice).
Interpersonal networks and collaboration at home and abroad are necessary at all stages of a global health experience (prior to departure, while in country, and upon return)	“I think the students who have been to the place before are definitely a big resource for people going for the first time, or even for the second time.” “But I think a way of doing it might be to actually do visit a country find colleagues that are working on something that you’re interested in, and see what skill set they are lacking and how you can sort of plug in or divert funding to achieving that aim. And isn’t that what global health is about? We’re sort of partnering with people abroad and plugging in holes that they can’t fill versus sort of setting the priorities for research or for intervention that might not be the same priorities your colleagues and your partner institutions have.” “But if there was something set up so that those of us returning from certain areas could kind of meet to talk about it and then even think about ways to continue the sustainability of our various projects and just brainstorm about that, because we all kind of came back and school started and life got busy and I personally thought it would have been really nice to have some sort of support group, maybe student-centered, to continue those thoughts and those efforts that we had spent so long working on” “I think the message that we should be sharing is how do we work together as global citizens.”	“Most students need significant framing of their experience and where they fit in a much longer story (i.e., the work doesn’t start when you arrive, or end when you leave) — so relates to managing expectations reframing how you define success, and working to build trust and relationships as the basis for any good work. Which takes patient listening – a skill that is not a natural common one among many American students.”
One major pitfall in global health work is a lack of appreciation of power dynamics (e.g., assigning priority to a student’s project over local needs and priorities)	“And I think one of the issues is that global health aims to be different from international health and from colonial medicine, and yet we’re entering a place where there’s such incredible power dynamics in terms of money and who has the skill set and the research and the wherewithal to go through certain projects.”	“Getting them [students] to embrace the reality that they will make little contribution in regards to impact through short duration student projects is important in managing expectations. Ideas that they will ‘change the world’ should be tampered with pragmatism.” [Students have] “myopic thinking.” “They’re [students] always expecting that they can solve health care issues in other countries because they’re smart and passionate.”
	“Oftentimes, people came to a place and sort of bull dozed over people that have lived there all their lives and really knew the problems that were there. They didn’t take the time to shake people’s hands and really put themselves in a position where they are a visitor and are being allowed to do work in a certain place.”	“No amount of academic prep can truly prepare you for field work.” “They don’t realize how much administrative work and human resource management is required for making a difference.” “They are not aware of the dangers of global health work.” Lack of recognition that initial global health experiences are mostly about education for the student and less about impact for the patient population. “Marrying the health care needs of individuals and populations in resource-limited countries with the most evidence-based methods of meeting those needs should be at the heart of all global health endeavors. Unfortunately, this is not always the case currently or historically. Global health endeavors should always be undertaken with the yardstick of how well a particular effort meets this standard and must always be delivered with cultural sensitivity, gaining a true understanding of the challenges of providing healthcare in challenging global settings, and the real ways that”
Bias exists everywhere and in multiple forms; students need to know their biases and be aware of others’ biases and address them whenever possible	“You start to stereotype…make judgments…start to say, ‘Oh, I know a little bit so that will then enable me to bridge the divide that pre-exists within the situation,’ You can always ask a question rather than jumping to a conclusion.” “So you see providers treating people differently and then people don’t want to go to these clinics and… just like within different groups, like I didn’t expect there to be so much mistrust, so much fear, so much racism because I haven’t been that way before.” “I did the semester abroad in XXX and guess ignorantly, I was thinking East Africa might be similar, like the places I would stay might look the same. The water access might look the same. Sanitation might look the same. So I was kind of expecting to really be grungy and rough-it, and then I get there and the apartment’s like nicer than my apartment here. There’s like great sanitation. Everything is very clean and very nice. That, to me, was a big slap in the face. I shouldn’t have just assumed that one country in Africa is very much like the other. And I was very happily surprised.” “Someone will assume that everyone in the room either feels the same way about something or thinks [everyone] has the same opinion about something or sees it the same way.”	“If students have not lived or worked in a low-income setting they usually are not well prepared to work there.” “[Students] do not understand how fragile settings are. Do not understand how our ‘exceptionalism’ is at times woven into our DNA – causing others to look at us with wary eyes and, causing us to behave in ways that are arrogant and clumsy.” “Students usually have a certain vision for what they think should happen, or perhaps what is best for another person”
Reverse culture shock and guilt about inequities can be pervasive	“You go to a developing world and see how people live their daily life with no expectation and then you come to a place like XXX, where people, for the most part mostly privileged compared to the rest of the world, and then you begin to see little things that we think are important. And I think for the first three months, I was like, ‘This is not important.’” “It’s really a drastic transition. You feel like you have to deprive yourself of many things to feel like an okay human being…”	
	“And never, ever felt prepared for the reverse culture shock…you spend so much time getting used to one standard of living and then find yourself like overwhelmed and disgusted and confused about the way the people live here…part of the reverse culture shock is then also how easily you go back to your normal life…what just happened, how did that experience just slide through my consciousness…I just feel like I never myself take time enough to reflect and kind of process what happens.” “I had situations where I felt conflicted not eating what everyone else was eating… I find that we in that setting just complain a lot about the food that people eat on a daily basis…” “I certainly had zero tolerance for a lot of things that people complain about here. And I would get mad about things just like you couldn’t tolerate.	
Ethical challenges are prevalent	“When is it human rights and when is it just culture, and when is it your place to question it.” “It never hurts to put yourself in someone else’s shoes. Like flip the situation on its head.” “…they used the language barrier between themselves and the patients as like an opportunity to talk freely in front of the patients without knowing that they couldn’t understand even in the context of as they were translating for a doctor, just like making jokes and kind of like, you know, judging what the patient would say as they were translating. That was a big part of it. That really bothered me. And the other part was knowing that there was no other healthcare in the area, kind of using it as a learning opportunity for themselves… ‘Oh, I can try pulling teeth because there’s no regulations out here’.” So using it more as an experience for themselves rather than the best care possible for the people that we were seeing…” “I actually got a really bad reaction when I would ask people for a signature, for a thumbprint or even for like a verbal okay, because in the area that I was in, a lot of people had been coming around and asking them for signatures to relocate that village and so I was… actually, someone screamed at me, yelling at me, telling me that… because they thought that I was part of that and they thought that I was taking advantage of them by getting a signature for like whatever with my motives. And so, it was just a very, very delicate way to get consent without making them feel like I was taking advantage of them.” “… the ethics of identifying problems kind of like forming relationships with the community and what your responsibility is as far as continuity with that is. Especially with identifying problems. Like seeing things that you’re not able to solve, but you could solve potentially but… I don’t know. It’s just a hard situation. I would have liked to … have an action plan like how to address some of the things that came up when I was there.” “I think communication is extremely important… People underestimate how important and how challenging it is, and I think a huge part of that is language, and culture also. But I think that for whatever reason, when you can’t speak someone else’s language, there’s a tendency to forget how to communicate, and I think that maybe the rules of polite conversation don’t necessarily apply because you can’t speak the same words as them.”	

**Figure 1:**
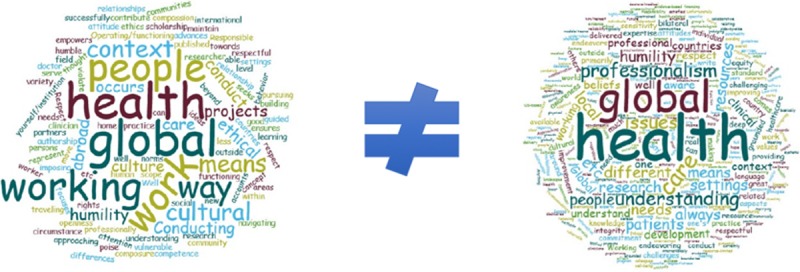
Word Cloud Depiction of Student and Faculty Open-Ended Responses.

Using a feature of the Qualtrics survey platform, we created a word cloud graphic from the student (left) and faculty mentor (right) open-ended survey responses. The size of each word in the clouds is weighted based on the frequency it appeared, i.e., the larger the word in the graphic, the more often respondents used it in their survey responses. This step allowed for additional quantitative evaluation of their qualitative survey responses (Figure 1).

## Discussion

This multi-modal study at a large academic institution reveals a number of key perspectives and skills that trainees and mentors recommend to effectively prepare for work abroad and prevent or mitigate challenges during their STEGH.

One of the resounding themes captured in this study is that global health experiences are fortified by and likely to result in enhanced benefit to both in-country staff and the population served when trainees practice humility, respect, and flexibility. A conflated belief in one’s ability could result in unintended consequences or harm to the local populace. Both trainees and mentors stated that training should instead foster key personal attributes. As one trainee said, “I think the message that we should be sharing is how do we work together as global citizens.” A mentor’s observation elaborates on the “how:” “[Global health professionalism involves a] deep appreciation of BOTH the values, attitudes, beliefs, and resources of other social groups and communities AND the boundaries and potential impact (both positive and negative) of one’s own values, attitudes, beliefs, and resources.”

An important study finding is that, while trainees felt they were prepared or extremely prepared for their global health experiences, a majority of mentors believed that trainees were actually unprepared or very unprepared. As one mentor said, “Most students need significant framing of their experience and where they fit in a much longer story…” Incorporating the expertise of local, on-site mentors by inviting them to co-teach in global health trainings, be lead protagonists in project design and implementation, and key authors on manuscripts and other deliverables, is vital to trainee preparation and overall learning experience [12].

The most prominent challenges faced by trainees working abroad were confronting bias and guilt about pervasive inequities and addressing ethical considerations (e.g., informed consent, power dynamics). An intriguing finding was that only a small percentage of learners wanted more training in ethics and human rights prior to their STEGH, even though the majority said trainees were very likely to encounter an ethical dilemma, half of our student respondents experienced an ethical dilemma, and half of these felt unprepared to address it. Trainees reported that there were unforeseen biases about the local region, culture, and practices by both trainees and faculty members. One trainee reflected, “I didn’t expect there to be so much mistrust, so much fear, so much racism.” Faculty shared the sentiment that trainees were often ill-prepared to face the challenges posed by working in an unfamiliar setting because of the perceived or stated expectations set prior to their departure. One had this to say: “Students usually have a certain vision for what they think should happen, or perhaps what is best for another person.”

A final key activity that survey participants desired is creating longitudinal, meaningful interpersonal networks and collaboration at home and abroad among their peers, mentors, and on-site colleagues at all stages of a global health project: prior to departure, while in country, upon return, and for the foreseeable future. Trainees were critical of one-time, short-term experiences, which they stated are more extractive and unilateral (mostly or only beneficial to the visiting trainee) and create a substandard system of care for populations with significant health and other needs. Since longitudinal engagement is a well-known best practice, further efforts in developing systems for long-term partnerships is needed [19].

There is a tension in STEGHs between benefits and unintended consequences to the in-country institutions and local populace. As the faculty mentors reported, we are challenged to balance the potential benefit of trainees’ enthusiasm with the potential pitfall of siphoning time, resources, and energy away from the local staff and other prioritized work.

The word clouds provide further insight. In general, while the terms ‘global’ and ‘health’ were among the most prominent words visually in both word clouds, there are notable thematic differences. The words ‘working’, ‘work’, ‘conducting’, and ‘projects’, are visually represented in the student word cloud. This implies student responses frequently related to actively doing something during their experience abroad. Comparatively, the words ‘humility’, ‘professionalism’, and ‘understanding’ are visually represented in the faculty mentor word cloud. This suggests that faculty are interested in qualities related to student learning and professional growth in addition to, or more so, than students *doing* something during their STEGH. These sentiments are supported by survey results, where faculty felt students were not adequately prepared for their STEGH, while students felt they were. Both word clouds share the words ‘humility’ and ‘people’. These findings may illustrate that while both students and mentors agree that people in the communities are at the center of these educational activities, and that this requires humility on the part of learners, the overall narrative for students seems more focused on a tangible deliverable of some sort as compared to skills and attitudes, such as professionalism, that can be cultivated through long-term experiences. These differences may also help explain why students felt they were prepared and faculty felt they were not. Students felt they had the concrete skills to complete a project of some sort while on site, while faculty may have felt students needed deeper knowledge and understanding of the local context and how their STEGH fit within local staff and community-defined priorities.

In addition to advocating for bidirectional, longitudinal, engagement, one potential solution to help trainees effectively prepare for work abroad and prevent or mitigate ethical dilemmas during their STEGH is to enrich existing training and ongoing mentorship to include the following: leadership, diplomacy, and project management; bias and ethics; lifelong interpersonal networks and collaboration. Academic institutions are developing clinical global health courses that introduce trainees to essential skills such as Helping Babies Breathe, bag-mask ventilation, and basic ultrasound [4,10]. However, acquisition of these types of skills is only one aspect of training and insufficient if our goal is to have well-rounded trainees who will be lifelong learners and leaders. Authors BS and JK are in the process of creating a course in global health professionalism that begins with learning labs where students have begun to debate ethical challenges. Author BS has spearheaded the implementation of a comprehensive global oral health competency-based training curriculum that beings to incorporate this study’s recommendations [13]. Other institutions have created high-fidelity global health ethics simulations [2].

In addition to promoting bi-directional, longitudinal engagement and robust pre-departure ethical preparation, a potential solution to bridge the gap between the perceptions of trainees and mentors is to clearly set the goals and expectations of students in the context of the local population’s needs and frequently review and reflect on these when participating in STEGHs. Providing students a well articulated outline of their purpose and an open dialogue about the common and frequently encountered challenges may broaden their perspective toward understanding that they are the more likely beneficiaries of the experience rather than the population of patients they treat.

This study has limitations. The study population is from only two institutions at the same university – a large medical school and a small dental school – and is not necessarily representative of all trainees and mentors who engage in global health work. It was voluntary and we had a relatively modest response rate, which may have resulted in selection bias. Focus group responses may have been tempered because of unspoken power dynamics between the faculty who acted as focus group facilitators and the learners. Trainees may not have been fully transparent and forthright because of fears of how they would be portrayed. This may reflect social desirability bias. Investigators attempted to mitigate this potential bias by explaining the purpose and confidential nature of the study and that all responses were encouraged. Finally, both the focus group and online survey responses are subject to recall bias.

## Conclusion

Just as interest and participation in global health work has grown significantly, so too have opportunities to enhance training based on ever-growing evidence of what works and what is feasible, permissible, and appropriate in diverse global health settings, especially resource-constrained ones. This multi-modal study reveals competencies and skills that require more intensive, ongoing training, including leadership, interpersonal and communication skills, project management, and ethics. In a globalizing world, there is a new obligation for all trainees to know how to apply skills to a global health setting, learn new ones that are germane for health care delivery in resource-constrained settings, and empower local staff and colleagues (under appropriate guidance and not beyond one’s capability) to promote capacity-building with the ultimate goal of sustainability. With continued interest and effort, we can cultivate our trainees to improve the health and well-being of their patients long past the duration of their global health experiences.

## Additional File

The additional file for this article can be found as follows:

10.5334/aogh.2721.s1 Appendix A. Focus group guiding questions.

## References

[1] Association of American Medical Colleges (AAMC). Association of American Medical Colleges: Medical School Graduation Questionnaire. 2018 Accessed 2019 Aug 22. aamc.org/download/490454/data/2018gqallschoolssummaryreport.pdf.

[2] Asao S, Lewis B, Harrison JD, et al. Ethics Simulation in Global Health Training (ESIGHT). MedEdPORTAL. 2017; 13: 10590 DOI: 10.15766/mep_2374-8265.10590 30800792 PMC6338194

[3] Benzian H, Greenspan JS, Barrow J, et al. A competency matrix for global oral health. J Dent Educ. 2015; 79(4). 25838005

[4] Butteris SM, Gladding SP, Eppich W, Hagen SA, Pitt MB, SUGAR Investigators. Simulation Use for Global Away Rotations (SUGAR): preparing residents for emotional challenges abroad: a multicenter study. Acad Pediatr. 2014; 14(5): 533–41. DOI: 10.1016/j.acap.2014.05.004 25169165

[5] Doobay-Persaud A, Chuang CJ, Evert J. Global health pedagogy: The art and science of teaching global health. Global Health Experiential Educ. 2017: 11–11. DOI: 10.4324/9781315107844-2

[6] Drain PK, Holmes KK, Skeff KM, Hall TL, Gardner P. Global health training and international clinical rotations during residency: Current status, needs and opportunities. Acad Med. 2009; 84: 320–325. DOI: 10.1097/ACM.0b013e3181970a37 19240438 PMC3998377

[7] Elansary M, Graber LK, Provenzano AM, Barry M, Khoshnood K, Rastegar A. Ethical dilemmas in global clinical electives. J Glob Health. 2011; 1(1): 24–27.

[8] Jeffrey J, Dumont RA, Kim GY, Kuo T. Effects of international health electives on medical student learning and career choice: Results of a systematic literature review. Fam Med. 2011; 43(1): 21–8. 21213133

[9] Lu PM, Park EE, Rabin TL, et al. Impact of Global Health Electives on US Medical Residents: A Systematic Review. Ann Glob Health. 2018; 84(4): 692–703. DOI: 10.29024/aogh.2379 30779519 PMC6748170

[10] Nelson BD, Saltzman A, Lee PT. Bridging the global health training gap: Design and evaluation of a new clinical global health course at Harvard Medical School. Med Teach. 2011; 34(1): 45–51. DOI: 10.3109/0142159X.2011.577122 21592020

[11] Peluso MJ, Kallem S, Elansary M, Rabin TL. Ethical dilemmas during international clinical rotations in global health settings: Findings from a training and debriefing program. Med Teach. 2018; 40(1): 53–61. DOI: 10.1080/0142159X.2017.1391374 29094625

[12] Roebbelen E, Dorman K, Hunter A, Kraeker C, O’Shea T, Bozinoff N. “They Will Come to Understand”: Supervisor Reflections on International Medical Electives. Teach Learn Med. 2018; 30(4): 377–385. DOI: 10.1080/10401334.2018.1437040 29565733

[13] Seymour B, Shick E, Chaffee B, Benzian H. Going Global: Toward Competency-Based Best Practices for Global Health in Dental Education. J of Dental Educ. 2017; 81(6): 707–715. DOI: 10.21815/JDE.016.034 28572417

[14] Stapleton G, Schroder-Back P, Laaser U, Meershoek A, Popa D. Global health ethics: An introduction to prominent theories and relevant topics. Glob Health Action. 2014; 7: 23569 DOI: 10.3402/gha.v7.23569 24560262 PMC3925811

[15] Thompson MJ, Huntington MK, Hunt DD, Pinsky LE, Brodie JJ. Educational effects of international health electives on U.S. and Canadian medical students and residents: A literature review. Acad Med. 2003; 78(3): 342–7. DOI: 10.1097/00001888-200303000-00023 12634222

[16] Tiller R, Jones J. Ethical reflection for medical electives. Clin Teach. 2018; 15(2): 1 DOI: 10.1111/tct.12657 28493360

[17] Harrison JD, Logar T, Le P, Glass M. What are the ethical issues facing global-health trainees working overseas? A multi-professional qualitative study. Healthcare. 2016; 4(3): 43 DOI: 10.3390/healthcare4030043 PMC5041044 27417631

[18] Tubman M, Maskalyk J, Mackinnon D, et al. Tackling challenges of global health electives: Resident experiences of a structured and supervised medicine elective within an existing global health partnership. Can Med Educ J. 2017; 20; 8(2): e4–e10. PMC5669288 29114341

[19] Wondimagegn D, Pain C, Baheretibeb Y, et al. Toronto Addis Ababa Academic Collaboration: A Relational, Partnership Model for Building Educational Capacity Between a High- and Low-Income University. Acad Med. 2018; 93(12): 1795–1801. DOI: 10.1097/ACM.0000000000002352 29995668 PMC6282678

[20] Doobay-Persaud A, Evert J, Decamp M, et al. Practising beyond ones scope while working abroad. The Lancet Glob Health. 2019; 7(8). DOI: 10.1016/S2214-109X(19)30291-8 31303286

