# Supplementary material for: Perspectives and Solutions from Clinical Trainees and Mentors Regarding Ethical Challenges During Global Health Experiences
Source: Ann Glob Health. 2020 Mar 26;86(1):34. doi: 10.5334/aogh.2721 (PMC7101005; doi:10.5334/aogh.2721)
Supplement: Appendix A. — Focus group guiding questions. [file agh-86-1-2721-s1.pdf]

Appendix A: Focus group guiding questions

1. Let's start the discussion by thinking about what makes a person globally competent. How would you describe a globally competent person?
2. How prepared were you for what you encountered during your summer global health project?
3. What aspects of your required preparation did you think was most useful?
4. What themes/issues do you wish you had more expertise in prior to engaging in your summer global health project?
5. How well-informed were you in the topics that were most relevant to your work? In what areas could you have been better informed?
6. Did you discover any biases or preconceived notions in yourself during this summer global health experience? If so, please describe.
7. Did you experience any culture shock? reverse culture shock? If so, how did you cope?
8. What was your greatest "takeaway" from this experience? Your greatest accomplishment?
9. What was your greatest challenge?
10. What recommendations do you have for future students? What topics would you like to see in the HMS/HSDM curriculum to address your identified gaps/challenges (pre, during and after)?
